# Determinants and willingness to practice obstetric analgesia among women attending antenatal clinic at Dr. Bogalech Gebre Memorial General Hospital Central Ethiopia: a cross-sectional study

**DOI:** 10.1186/s12884-024-06674-x

**Published:** 2024-07-10

**Authors:** Teketel Ermias Geltore, Getachew Alemu, Ayanos Taye, Eden Sileshi, Merkin Bekele, Lakew Lafebo Foto

**Affiliations:** 1 Department of Midwifery, College of Medicine and Health Science, Wachemo University, Durame Campus, Durame, Ethiopia; 2 Department of Medical Laboratory Science, College of Medicine and Health Science, Wachemo University, Durame Campus, Durame, Ethiopia; 3 Nursing Department, School of Nursing and Midwifery, College of Health Sciences and Medicine, Jimma University, Jimma, Ethiopia; 4 School of Public Health, Institute of Health Science, BuleHora University, BuleHora, Ethiopia; 5 Teketel Ermias Geltore, PO Box 667, Wachemo, Ethiopia

**Keywords:** Determinants, Willing, Antenatal care, Labor analgesia, Pregnant women, Ethiopia

## Abstract

**Background:**

Labor pain is uniquely experienced and described by the woman giving birth, and it is often considered one of the most excruciating experiences for many women. This study aimed to evaluate factors associated with the willingness to receive labor analgesia among women attending the antenatal clinic at Dr. Bogalech Gebre Memorial General Hospital Central Ethiopia in 2022.

**Methods:**

An institution-based, cross-sectional study was conducted from January to March 2022. Data were collected using semi-structured questionnaires by a convenience sampling technique. Data was entered in EpiData 4.2 and exported to SPSS version 20 for analysis. Both Bivariable and multivariable logistic regressions were conducted to determine factors associated with pregnant women’s willingness to choose labor analgesia. Crude odds ratio (COR) and adjusted odds ratio (AOR) were computed to assess the association between variables.

**Results:**

A total of 398 pregnant women have participated in the study with a response rate of 94%. Nearly 30%, (29.4%) of the pregnant women had a willingness to practice labor pain management. Being a housewife (AOR: 8.35, 95% CI: 2.07, 33.63). Women who live in urban (AOR: 2.60, 95% CI: 1.29, 5.29). Having had awareness about labor analgesia (AOR: 1.70, 95% CI: 1.00, 2.60) and the short duration of labor time (AOR: 1.84, 95% CI: 1.15, 2.96) were statistically significant with a willingness to practice labor analgesia.

**Conclusion:**

We conclude that the willingness of pregnant mothers’ toward obstetric analgesia practice was low in the study area. Being a housewife, urban residence, awareness about labor analgesia, and short duration of labor were statistically significant with the willingness of the mothers to practice labor analgesia. To increase willingness to use labor analgesia, authorities should prioritize delivering health education on pain management choices to address concerns and promote effective methods and practices.

**Supplementary Information:**

The online version contains supplementary material available at 10.1186/s12884-024-06674-x.

## Background

Labor-linked pain is one of the worst pains that the majority of women familiarity during giving birth [1–3]. Psychological and cultural factors possibly shape women’s individual experiences during childbearing [4]. Around the world, many women opt for cesarean section over vaginal delivery because of the physical pain they endure during labor [5–7]. A woman’s knowledge and confidence in handling labor pain can also impact her decision on pain relief methods during childbirth [8].

Factors such as parity, maternal age, culture, social status, ethnicity, maternal and fetal weight, fetal position, educational attainment, and having a companion during labor influence a parturient perception of labor pain [8–10]. The United Nations now recognizes pain management as a fundamental human right in health [11]. Despite this, concerns regarding pain relief and human rights remain in certain areas [12–15]. Women should feel free to choose whatever pain relief methods they want [8]. Many expectant mothers seek options for pain relief during childbirth due to concerns about the intensity of labor pain. Some women choose to manage the process naturally, while others opt for pharmacological methods like epidural anesthesia (EA) to make childbirth more manageable and less stressful [16, 17]. To achieve less mother distress and to facilitate the progress of labor, pain relief during childbirth is mandatory. In this regard, decrement of pain intensity needs suitable techniques, to manage pain during delivery [18].

The study’s results indicated that over 23% of primipara characterized this pain as excruciating, while 65% described it as strong and only 9% found it tolerable. Conversely, among multipara, only 17% considered this pain intolerable, 46% perceived it as strong, and 25% deemed it bearable. [19, 20].

Because of a lack of knowledge about ways to alleviate labor pain, expectant mothers suffer from the agony of labor pain and their uncontrollable anxieties about it [21–23].Research conducted in India showed that half of all cesarean deliveries were performed on women in labor because of their previous negative experiences with labor pain [24]. Misconceptions about the acceptability, safety, and availability of pain relief options are the primary factors why women in low- and middle-income countries do not receive adequate pain management [25].

Providing sufficient pain relief during labor is associated with increased satisfaction with the overall birth process and quality of maternity services [26]. However, in low and middle-income nations, healthcare providers frequently overlook the importance of addressing pain management during childbirth [25, 27–29].

Ethiopia’s government has faced challenges in enhancing the provision of labor pain relief methods, including physical, psychological, and pharmacological approaches and incorporating them as essential skills. However, the implementation process remains unclear, likely because stakeholders are not sufficiently focused on the issue [30–33].

**Fig. 1:**
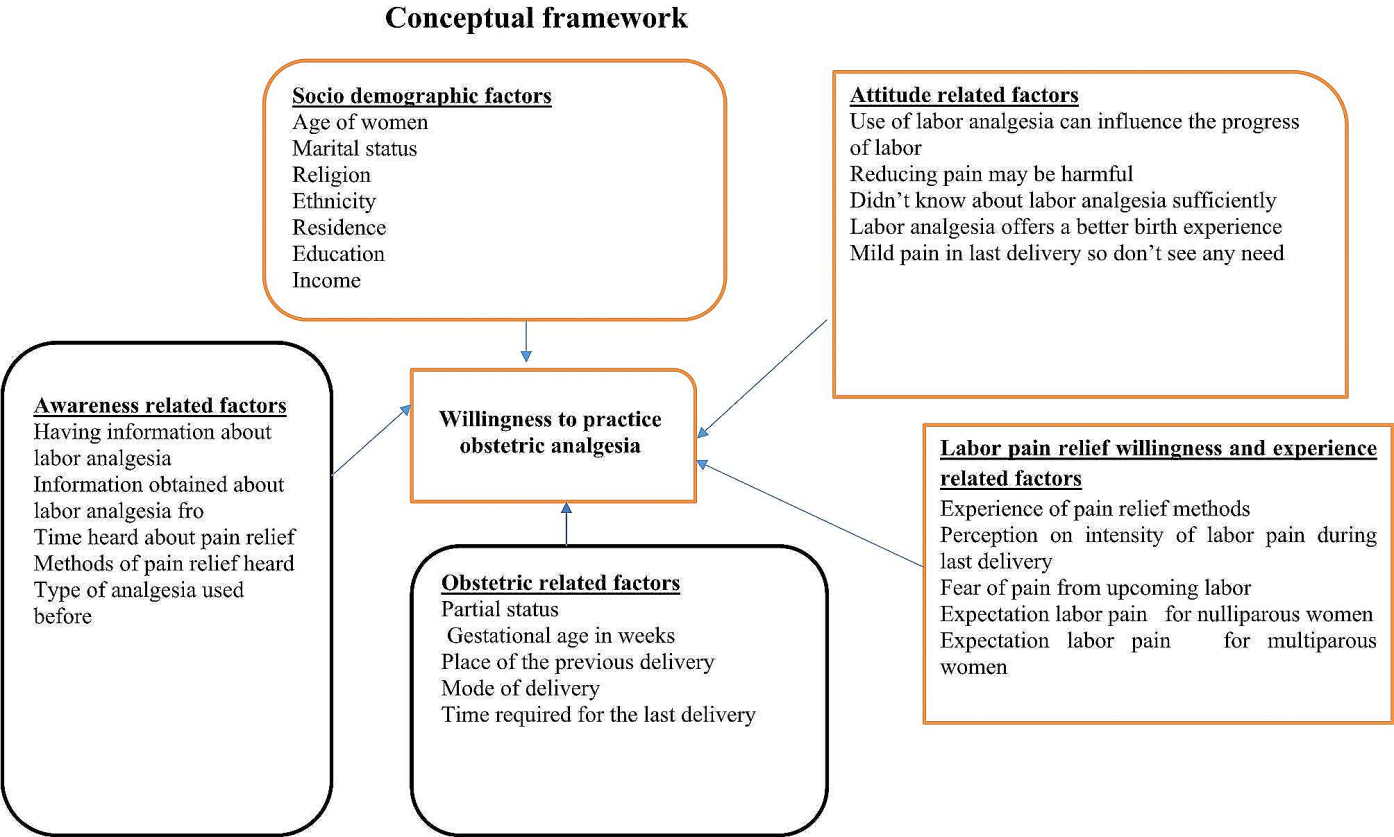
Conceptual frame work developed after reviewing different literatures

Findings from Ethiopia showed that the utilization of labor pain relief was approximately 40.1%, 43.3%, and 37.9% in Amahara, Tigray, and Central Ethiopia regions. Non-pharmacologic methods were commonly used for labor pain relief [33–35]. Past research primarily examined obstetric care providers, whereas this study concentrated on pregnant women experiencing labor pain and its associated issues.**(**Fig. 1) is a conceptual framework to illustrate how the outcome variable (willingness to practice labor analgesia) could be affected by various socio-economic, obstetric, perception related and pain expectation and experience related factors. It was constructed upon extensive reviewing of similar studies. Therefore, this research aimed to evaluate the readiness to utilize obstetric pain relief and the associated factors among women visiting the antenatal clinic at Dr. Bogalech Gebre Memorial General Hospital in Southern Ethiopia in 2022.

## Methods

### Study settings

Dr. Bogalech Gebre Memorial General Hospital is situated 274 km away from Addis Ababa, with a medical staff consisting of three general surgeons, one gynecologist, one internist, one pediatrician, nine health officers, 17 general practitioners, 29 BSc nurses, 41 clinical nurses, 23 midwives, and 2 integrated emergency surgery officers.

### Study design and data

The hospital’s monthly ANC follow-up rate averages 50–60 in 2021. The research, conducted from January to March 2022. Institutional-based quantitative cross-sectional study design was employed. All pregnant women who attended the ANC clinic at Dr. Bogalech Gebre Memorial Hospital during the study period.

### Eligibility

All pregnant women attending antenatal care at Dr. Bogalech Gebre Memorial Hospital during the study period and willing to take part were included, except for those < 18 years old, unable to communicate, or who declined to join.

### Sampling and sample size

The sample size for this study was calculated using a single population proportion formula by considering: a 95% confidence level, margin of error (0.05), *p* = 0.5, and by using the formula n= (Zα/2)2p (1-p)/d2.The estimated sample size, by using the above-mentioned formula and by considering a 10% non-response rate was 423. The study participants were selected using convenience sampling, where every consecutive pregnant woman was included until the desired sample size was achieved.

### Operational definitions

#### Pain

Pain levels were assessed through the Numerical Rating scale, where participants rated their pain from 0 to 10. The scale ranged from no pain (0) to severe pain [10], as displayed in Table 1.

**Table 1:** Displays the numerical rating scale utilized for pain assessment

No pain	0
Mild pain	1–3
Moderate pain	4–7
Severe pain	8–10

#### Willingness

Women agreeing to receive labor pain relief for their upcoming delivery [24, 36].

#### Experience

Nulliparous women had not given birth before, while parous women had at least one delivery [37].

#### Awareness

Women confirming prior awareness of labor pain relief [38].

#### Positive attitude

Pregnant women who answered greater than or equal to mean value of a three-point Likers scale questions were classified as having positive attitude [39].

### Data collection

Data were collected using a semi-structured and pretested, questionnaire prepared by adapting from different studies [5, 10, 14, 40]. The tools first were prepared in English and then converted to the local language (Kambatissa) and back to English again to check validity. The questionnaire had five essential components related willingness to practice labor analgesia among the pregnant women including providers’ Socio demographic characteristics of the study participants, obstetric related questions, labor pain relief willingness and experience related questions, awareness about labor analgesia methods related questions and attitude about labor analgesia practice related questions. Eight items were used to assess the socio-demographic characteristics of the study participants, five items used to assess obstetric characteristics of the study participants, six items were used to assess pain expectation and experience among pregnant women, five items were used to assess awareness and five items were used to assess attitude of labor analgesia methods among pregnant women attending antenatal care.

Five diploma midwives data collectors and two supervisors were assigned. One day training was given to the data collectors and supervisors, before actual data collection. The study participants were invited to participate after getting antenatal care service from the antenatal clinic and the interview was undertaken in a private room to maintain privacy which took 25–30 min for each individual.

### Measurements

Data was verified and inputted into Epidata version 3.1 before being exported to Statistical Package for Social Sciences version 20 for further analysis. Tables and graphs were utilized for data presentation. In binary logistic regression, potential factors associated with willingness to practice labor analgesia at *P* < 0.25 were recruited for the adjustment in the multivariable model. In multivariable logistic regression analysis, the adjusted odds ratio (AOR) with 95% CI was used to measure the strength of association, and variables with *P* < 0.05 were considered statistically significant. The model goodness of fit was checked by the Hosmer and Lemeshow test; when *P* > 0.05, the model fit the data reasonably better. Data quality was maintained throughout data collection, coding, entry, and analysis. The completeness of the collected data was checked daily by data collectors and supervisors. A 5% pretest was done at Shinshcho Primary Hospital outside of the study area before actual data collection. The internal consistency of attitude, knowledge, and practice questions was assessed using Cronbach’s alpha for reliability.

## Results

### Participation

The study included 398 pregnant women, with a participation rate of 94%. Majorities were aged between 25 and 30 years old (56.8%). Most were of Kembata ethnicity (95.5%) and Protestant religion (76.4%). The majorities were married (97%) and lived in urban areas (87.2%). Substantial portions were government employees (42.2%). Around a quarter could read and write (24.6%) and had low income (60.8%) as shown in Table 2.

**Table 2:** Shows the socio-demographic characteristics of the pregnant women willing to receive labor analgesia at antenatal clinic at Dr. Bogalech Gebre Memorial General Hospital, Central Ethiopia, 2022 (*N* = 398)

Variables	Category	Frequency	Percentage
Age in years	18–24	141	35.4
	25–31	226	56.8
	> 31	31	7.8
Marital status	Married	386	97.0
	Singe	8	2.0
	Others**	4	1.0
Religion	Protestant	304	76.4
	Orthodox	71	17.8
	Muslim	9	2.3
	Catholic	14	3.5
Ethnicity	Kembata	380	95.5
	Hadiya	6	1.5
	Wolaita	2	0.5
	Amara	9	2.3
	Gurage	1	0.3
Residence	Rural	51	12.8
	Urban	347	87.2
Education	Unable to read and write	70	17.6
	Can read and write	98	24.6
	Primary	88	22.1
	Secondary	87	21.9
	Graduated	55	13.8
Occupation	Housewife	14	23.9
	Merchant	121	42.2
	Governmental employee	168	30.4
	Farmer	95	3.5
Income	High income	161	38.1
	Low income	262	61.9

∗∗Others = divorced, married but living apart

### Obstetric characteristics of the study participants

Over half of the participants in the study had given birth more than once. Most participants (77.2%) were in their third trimester. The majority of previous deliveries (78.3%) took place in hospitals. For over half of the participants, their previous delivery method was a spontaneous vaginal delivery and the labor duration was *≤* 12 h (63.3%) as shown in Table 3.

**Table 3:** Obstetric characteristics of the pregnant women willing to receive labor analgesia at antenatal clinic of Dr. Bogalech Gebre Memorial General Hospital, Central Ethiopia, 2022 (*N* = 398)

Variable	Category	Frequency	Percentage
Partial status	Nulliparous	191	48.0
	Multipara	207	52.0
Gestational age in weeks	13–28	138	34.7
	> 29	260	65.3
Place of the previous delivery	At health centers	45	21.7
	At hospitals	162	78.3
Mode of previous delivery	Normal (spontaneous vaginal delivery)	111	53.6
	Other than normal	96	46.4
Time required for the last delivery	> 12 h	7 6	36.7
	*≤* 12 h	131	63.3

### Labor pain relief willingness and experience of the study participants

The participants in the study were asked about their willingness to use labor pain relief methods. 29.4% expressed a willingness to do so. 66.3% had not yet experienced these methods, with 50.7% finding labor intensity to be moderate. Over half, 53.3%, reported a high fear of pain during labor. Expectations of severe labor pain were reported by 72.9% of nulliparous women and 45.9% of multiparous women. Most participants, 61.0%, believed that labor pain should be managed as shown in Table 4.

**Table 4:** Pain expectation and experience among pregnant women receiving ANC at Dr. Bogalech Gebre Memorial General Hospital Central Ethiopia, 2022 (398)

Variable	Category	Frequency	Percentage
Experience of pain relief methods	Yes	134	33.7
	No	264	66.3
Perception on intensity of labor pain during last delivery	Mild	27	13.1
	Moderate	105	50.7
	Severe	75	36.2
Fear of pain from upcoming labor	Very high	212	53.3
	High	152	38.2
	No fear	34	8.5
Expectation labor pain for nulliparous women	Pain free	30	7.50
	Mild	10	2.51
	Moderate	22	5.53
	Severe	336	84.4
Expectation labor pain for multiparous women	Pain free	10	2.50
	Mild	75	18.8
	Moderate	130	32.8
	Severe	183	45.9
Do you believe labor pain should be managed	Yes	244	61.0
	No	154	39.0

### Awareness of labor analgesia methods among study participants

Most study participants did not know labor pain relief methods, with healthcare providers being a common source of information. Nearly half of the participants learned about pain relief methods during previous pregnancies. A significant portion heard about massage and deep breathing, with many using these techniques shown in Table 5.

**Table 5:** Awareness of labor analgesia methods among pregnant women receiving ANC at Dr. Bogalech Gebre Memorial General Hospital Central Ethiopia, 2022 (398)

Variable	Category	Frequency	Percentage
Having information about labor analgesia	Yes	57	34.1
	No	110	65.9
Information obtained about labor analgesia from	The media	10	5.9
	Antenatal talks in maternity and child health	28	16.8
	Friends	14	8.5
	Experience from previous delivery	55	32.9
	Health care provider	60	35.9
When did you heard about pain relief	During current pregnancy	35	8.9
	During previous pregnancy	79	47.4
	During previous childbirth	53	31.7
Methods of pain relief you heard about	Inhaled analgesia	2	1.22
	IV Pethidine or Morphine	23	13.8
	IM in the thigh shoulder buttock	14	8.4
	IV in the lower back	12	7.2
	Massage deep breathing	116	69.4
Which type of analgesia have you used before	IM Pethidine or Diclofenac	22	13.2
	IV Pethidine or Tramadol	17	10.2
	Massage deep breathing	128	76.6

### Attitude of labor analgesia methods among the study participants

Around 45% of participants were unsure about the impact of labor analgesia on labor progress, while nearly half were unaware of its potential to cause fetal distress. Similarly, a third of participants believed women should endure natural labor pain, but a similar number agreed that labor analgesia provides a more positive birthing experience. Additionally, over 40% of participants expressed concern about the possibility of needing instrumental delivery as shown in Table 6.

**Table 6:** Attitude of pregnant women towards labor analgesia methods at Dr. Bogalech Gebre Memorial General Hospital Central Ethiopia, 2022 (398)

Variable	Category	Frequency	Percentage
Use of labor analgesia can influence the progress of labor	Disagree	169	42.5
	Neutral	52	13.0
	Agree	177	44.5
Didn’t know about labor analgesia sufficiently	Disagree	145	36.8
	Neutral	194	49.2
	Agree	59	14.0
Reducing pain may be harmful	Disagree	128	31.5
	Neutral	135	34.3
	Agree	135	34.3
Labor analgesia offers a better birth experience	Disagree	106	25.8
	Neutral	132	33.5
	Agree	160	40.5
Mild pain in last delivery so don’t see any need	Disagree	147	37.5
	Neutral	78	18.7
	Agree	173	43.8

### Factors associated with willingness to practice obstetric analgesia

Factors like age, occupation, residence, time for last delivery, awareness, and attitude were significantly linked to willingness for obstetric analgesia among women in antenatal clinics as shown in bivariate logistic regression analysis. Multivariable regression analysis indicated that being a farmer, living in urban areas, awareness, and shorter labor duration were associated with higher willingness for labor analgesia. Specifically, housewives were 8.4 times more likely to practice obstetric analgesia compared to government employees, urban residents were 2.62 times more willing than rural residents, those aware of obstetric analgesia were almost 2 times more willing, and women with shorter labor were 1.84 times more willing than those with longer labor as showed in Table 7. Age and from the category of occupation being farmer were not statistically significant with willingness to practice labor analgesia.

**Table 7:** Factors associated with willingness to practice obstetric analgesia among women attending antenatal clinic at Dr. Bogalech Gebre Memorial General Hospital Southern Ethiopia, 2022 (*n* = 398)

Variables		Willingness to practice obstetric analgesia		COR (95% C.I)	AOR (95% C.I)
		No	Yes		
Age	18–24	120	21	1	1
	25–31	106	120	6.47(1.78,3.31)*	2.21(0.98,3.88)
	> 31	19	12	4.24(0.41,4.96)	2.31(0.94,3.26)
Occupation	Governmental employee	147	21	1	1
	Merchant	90	31	2.41(1.94,4.43)*	0.77(0.42,1.43)
	Farmer	76	16	1.42(2.32,9.08)*	2.37(0.97,5.80)
	Housewife	6	8	9.33(2.81,28.07)*	8.35(2.07,33.63)*
Residence	Urban	221	126	4.21(2.74,6.64)*	2.62(1.29,5.29)*
	Rural	15	36	1	1
Awareness	Have no awareness	58	52	1	1
	Have awareness	21	36	1.91(1.1,2.5)*	1.7(1.0,2.6)*
Time required for the last delivery	> 12 h	45	31	1	1
	*≤* 12 h	42	89	3.07(2.03,4.58)*	1.84(1.15,2.96)*
Attitude	Had no positive attitude	74	189	1	1
	Had positive attitude	93	42	0.17 (0.30,0.93)*	0.59(0.19, 1.79)

*Note*^a^ statistically significant in COR: P-value < 0.25. ^b^ statistically significant in AOR: P-value < 0.05. Abbreviations: AOR, adjusted odds ratio; COR, crude odds ratio

## Discussion

This study has shown that the prevalence of willingness of pregnant women to practice labor analgesia was 29.4%. The willingness of pregnant women to practice labor analgesia was associated with being a farmer, living in urban areas, awareness about labor analgesia, and shorter labor duration. These findings are not encouraging, especially when it is expected that women should be willing, among other things, effective pain relief for their upcoming delivery. The finding suggests the need to emphasize strategies that help to improve the practice of labor analgesia among pregnant women during their upcoming delivery. These could be awareness creation about the service, availability and accessibility of the services, counseling about the complication associated with prolonged labor and significances of labor analgesia overall childbirth experience. The finding of this study was higher than the reported prevalence from a cross-sectional study in India which was 10.2% [41]. However, the finding of current study was lower than the proportion of willingness of pregnant women towards labor analgesia reported from cross sectional survey, Gorakhpur-India, which was 68% [36], an institution-based, cross-sectional study, Ethiopia which was 65.9% [42] and university teaching hospital in southern Nigeria, which was 81.4% [5] The variation observed in the proportion of willingness to practice labor analgesia among the pregnant women could be due to stem from better education on pain relief techniques in pregnancies and the socioeconomic status of the Indian population, awareness of the benefit of labor analgesia methods, myths and misconceptions related to the service.

In this study, being housewives were almost nine times more likely willing to practice labor analgesia as compared to government employees This finding was contrary to the results of studies conducted in Jimma medical center (JMC), Southwest Ethiopia [43], University of Gondar Comprehensive Specialized Hospital (UGCSH), Ethiopia [42]. However, the occupational category and population included in the study vary across studies. In the Southwest Ethiopia study, the population was those who had women who gave birth at JMC, and in respect to occupational status being farmer was negatively associated with labor pain management. In UGCSH, occupationally being housewife was positively associated with attitude of women towards labor analgesia. In current study, there was significant association between occupationally being housewife and willingness to practice labor analgesia among pregnant women when compared to government employees. The reason behind this might be the cultural belief among farmers that an inability to tolerate labor pain is a sign of emotional weakness and those women should cope with labor pain [42]. On the other hand, government workers may be more knowledgeable about pain-free childbirth through cesarean delivery, with private maternity clinics offering cesarean services. Research indicates an increasing cesarean rate in Ethiopia, [44], possibly due to government employees opting for cesarean birth to avoid labor pain.

Pregnant women who residing in urban areas, were almost three times more likely willing to practice labor analgesia than women living in rural area. Pregnant women who live in urban might have awareness about labor analgesia from various sources, so they have tendency to practice labor analgesia. This outcome aligns with the research carried out at Debre Tabor Specialized Referral Hospital [45].

The research findings indicated that women who were aware of obstetric labor analgesia were nearly twice as likely to consider practicing labor analgesia compared to others, aligning with similar results in India [36]. Having awareness may help them improve usage of adequate analgesia, which may in turn improve quality of care during labor and better outcome of mother and baby.

Additionally, women with shorter labor durations were 1.84 times more likely willing to practice labor analgesia compared to those with longer labor times. This study consistent with studies from the Netherlands [46]. This might be due to if duration of labor is prolonged it can result in loss of fetus, psychological trauma and other permanent complications.

## Conclusion

This study more than one-fourth of pregnant women had willingness towards obstetric analgesia. Factors like being a housewife, urban residence, awareness about labor analgesia, and short labor duration were found to be significantly associated with willingness for labor analgesia among pregnant women. Concerns about harmful effects, influence on labor progress, lack of sufficient knowledge, and mild pain in previous delivery were key reasons not to practice labor analgesia. Overall, pregnant women had inadequate awareness of obstetric analgesia methods, suggesting a need for improved health education on labor pain management to address concerns and misconceptions as well as enhance practice. It is suggested that adequate information should be given by antenatal service care providers to educate pregnant women about the benefits, modalities, and impediments of effortless labor. There should be a mass media campaign for demonstrating data about labor pain management. Leaflets containing points of interest on provision of labor analgesia amid antenatal visits can be another choice for decreasing the burden of educating about labor analgesia.

### Limitations

This was conducted at a single hospital in consecutive patients coming to the antenatal clinic therefore it may not be indicative of the entire population. Additionally, there was a discrepancy between the required sample size and the sample size obtained could be due to attributed to a significant decrease in contact coverage from the initial ANC visit to the fourth ANC visit. Moreover, this research did not include mothers in labor, potentially impacting the need for labor pain relief.

### Electronic supplementary material

Below is the link to the electronic supplementary material.

Supplementary Material 1

## Data Availability

Data will be available from the corresponding author on reasonable request.
