# Supplementary material for: Determinants and willingness to practice obstetric analgesia among women attending antenatal clinic at Dr. Bogalech Gebre Memorial General Hospital Central Ethiopia: a cross-sectional study
Source: BMC Pregnancy Childbirth. 2024 Jul 10;24:470. doi: 10.1186/s12884-024-06674-x (PMC11238436; doi:10.1186/s12884-024-06674-x)
Supplement: Supplementary file 1 — Supplementary Material 1 [file 12884_2024_6674_MOESM1_ESM.docx]

# Annex: 1 Questionnaires

**Part I: Socio-Demographic Characteristics of Respondents**

**Instruction: Please circle the number of your choice.**

| It. no. | Questions | Responses | Remark |
| --- | --- | --- | --- |
| 101 | How old are you? | ------------years |  |
| 102 | What is your marital status? | 1. Married 2. Single  3. Others (specify)__________ |  |
| 103 | What is your religion? | 1. Protestant 2. Orthodox  3. Musilm 4. Catholic  5.Others(specify)___________ |  |
| 104 | What is your ethnicity? | 1. Kembata 2. Hadiya  3. Wolaita 4. Amara 5. Gurage |  |
| 105 | Where is your residence? | 1. Rural 2. Urban |  |
| 106 | What is your level of qualification? | 1. Unable to read and write 2. Can read and write  3. Primary 4. Secondary 5. Graduate |  |
| 107 | What is your occupation? | 1 Housewife 2. Merchant 3. Governmental employee 4. Farmer |  |
| 108 | How much is your income? | 1. High income 2. Low income |  |

**Part II: Obstetric characteristics of women who visited the antenatal care clinic-related questions**

**Instruction: Please circle the number of your choice**.

| Item No. | Items | Response |
| --- | --- | --- |
| 201 | What is your parity? | 1.Nulliparous  2. Multipara |
| 202 | How long is Gestational age in weeks? | 1. 13-282. 2. >29 |
| 203 | Where was your place of the previous delivery?. | 1. At health centers 2. At hospital |
| 204 | What was your previous mode of delivery? | 1.Normal (spontaneous vaginal delivery)  2.Others |
| 205 | What time required for the last delivery? | 1. >12hours  2. < 12 hours |

**Part III: Pain expectation and experience -related questions Instruction:**

**Please click the box you choose**

| Item No. | Items | Response |
| --- | --- | --- |
| 301 | What is your parity? | 1. Yes 2. No |
| 302 | Perception on intensity of labor pain during last delivery | 1. Mild 2. Moderate 3. Severe |
| 303 | Fear of pain from upcoming labor | 1. No fear 2. High 3. Very high |
| 304 | Expectation labor pain for nulliparous women | 1. Pain free 2. Mild 3. Moderate 4. Severe |
| 305 | Expectation labor pain for multiparous women | 1. Pain free 2. Mild 3. Moderate 4. Severe |
| 306 | Do you believe labor pain should be managed | 1. Yes 2. No |

**Part IV: Awareness of labor analgesia methods -related questions Instruction:**

**Please click the box you choose**

| Item No. | Items | Response |
| --- | --- | --- |
| 401 | Having information about labor analgesia? | 1. Yes 2. No |
| 402 | From where did you obtain the information about labor analgesia from? | 1. The media 2. Antenatal talks in maternity and child health 3. Friends 4. Experience from previous delivery 5. Health care providers |
| 403 | When did you heard about pain relief? | 1. During this pregnancy 2. During previous pregnancy 3. During previous childbirth |
| 404 | Methods of pain relief you heard about? | 1. Inhaled analgesia 2. IV Pethidine or Morphine 3. IM in the thigh shoulder buttock 4. IV in the lower back 5. Massage deep breathing |
| 405 | Which type of analgesia have you used before? | 1. IM Pethidine or Diclofenac 2. IV Pethidine or Tramadol 3. Massage deep breathing |

**Part V: Attitude of labor analgesia method-related questions Instruction:**

**Please click the box you choose**

| Item S.No. | Questions | Response | | |
| --- | --- | --- | --- | --- |
|  |  | Disagree | Neutral | Agree |
| 531 | Use of labor analgesia can influence the progress of labor |  |  |  |
| 502 | Didn’t know about labor analgesia sufficiently |  |  |  |
| 503 | Reducing pain may be harmful |  |  |  |
| 504 | Labor analgesia offers a better birth experience |  |  |  |
| 505 | Mild pain in last delivery so don’t see any need |  |  |  |
